# Extrachromosomal circular DNA: a new potential role in cancer progression

**DOI:** 10.1186/s12967-021-02927-x

**Published:** 2021-06-10

**Authors:** Tianyi Wang, Haijian Zhang, Youlang Zhou, Jiahai Shi

**Affiliations:** 1 Nantong Key Laboratory of Translational Medicine in Cardiothoracic Diseases, and Research Institution of Translational Medicine in Cardiothoracic Diseases, Affiliated Hospital of Nantong University, No. 20, Xisi Road, Nantong, 226001 Jiangsu China; 2 Department of Thoracic Surgery, Affiliated Hospital of Nantong University, No. 20, Xisi Road, Nantong, 226001 Jiangsu China; 3 Research Center of Clinical Medicine, Affiliated Hospital of Nantong University, No. 20, Xisi Road, Nantong, 226001 Jiangsu China

**Keywords:** Extrachromosomal circular DNA, Molecular mechanisms, Cancer progression

## Abstract

Extrachromosomal circular DNA (eccDNA) is considered a circular DNA molecule that exists widely in nature and is independent of conventional chromosomes. eccDNA can be divided into small polydispersed circular DNA (spcDNA), telomeric circles (t-circles), microDNA, and extrachromosomal DNA (ecDNA) according to its size and sequence. Multiple studies have shown that eccDNA is the product of genomic instability, has rich and important biological functions, and is involved in the occurrence of many diseases, including cancer. In this review, we focus on the discovery history, formation process, characteristics, and physiological functions of eccDNAs; the potential functions of various eccDNAs in human cancer; and the research methods employed to study eccDNA.

## Introduction

As a type of DNA molecule, circular DNA is ubiquitous in nature and includes bacterial plasmids as well as some viral genomes [1–3]. More than half a century ago, researchers found that there is also a special class of circular DNA in eukaryotes, which is isolated from the normal genome, free from the chromosomal genome and participates in physiological or pathological processes in special ways [4, 5]. Because these DNA molecules exist independently from the chromosome and are often circular, they are called extrachromosomal circular DNAs (eccDNAs). However, research on eccDNA has been stagnant for a long time, and its biological function remains unknown due to the lack of suitable technology. However, in recent years, relying on the development of high-throughput sequencing technology and other research methods, researchers have achieved a new understanding of the structure and biological function of eccDNA.

There has always been confusion and a lack of clarity around the naming and classification of eccDNA, which has posed a problem for researchers. Since the size of eccDNA remains inconclusive and the mainstream view is that its length ranges from 10 to millions of bp (base pairs), this review classifies it into the following four categories based on size and sequence: (1) small polydispersed circular DNA (spcDNA) (100–10 kb) [6, 7], (2) telomeric circles (t-circles) (multiples of 738 bp) [8], (3) microDNA (100–400 bp) [9, 10], and (4) extrachromosomal DNA (ecDNA) (1–3 Mb) [11] (Table 1 [12]). Recent studies have shown that eccDNA is associated with a variety of diseases, such as ageing and cancer [13]. This review focuses on the potential role of eccDNA in cancer progression.

**Table 1:** Classification of eccDNA

Name of the eccDNA	Size	Characteristic	Function
spcDNA	100–10 kb	Homologous to repetitive chromosomal sequences; byproducts or intermediates of gene rearrangement	Initiation and enhancement of genomic instability
telomeric circles	Integral multiples of 738 bp	Duplex or single-stranded molecule composed of telomeric repeats	Involved in the ALT of telomeres
microDNA	100–400 bp	Origin from regions with high GC content and exon density	Expressing functional small regulatory RNAs, including microRNAs and new si-like RNAs
ecDNA	1–3 Mb	Acentric, without telomere, containing full genes	Amplifying genes related to carcinogenesis and drug resistance

## The discovery of eccDNA

The discovery of eccDNA dates back to 1965. Hotta et al. identified circular DNA outside chromosomes in wheat embryos and boar sperm [4]. In the same year, Cox D et al. found extrachromosomal DNA in malignant tumours of childhood [5]. Because this type of DNA often appeared in pairs, it was also referred to as “double minutes” (DMs). At the same time, researchers found many examples of polydispersed circular DNA of different sizes in boar sperm, Bacillus megaterium and yeast cells, which was later identified in HeLa cells and officially named small polydispersed circular DNA (spcDNA) [4, 14–18]. Thereafter, researchers discovered eccDNA in many species, such as yeast, and in mammals and plants [19–21]. With the development and maturation of molecular cloning technology, researchers began to study the molecular function of eccDNAs. In 1978, Alt FW et al. found DMs carrying the dihydrofolatereductase (DHFR) gene in methotrexate-resistant mouse cells [22]. The existence of these double microbodies led to amplification of the DHFR gene and mediation of the resistance of mouse cells to methotrexate, and this was the first time that the biological effects associated with DMs were reported. In 1983, Kohl NE et al. reported the existence of DMs carrying N-MYC in neuroblastoma [23]. Since then, a number of studies have determined that DMs can carry oncogenes, including epidermal growth factor receptor (EGFR) and c-MYC [24]. At the same time, researchers have also found that DMs often exist in the form of circular DNA and that they account for only approximately 30% of extrachromosomal DNA [24]. Therefore, the term DMs was gradually replaced by the expression eccDNA.

The completion of the Human Genome Project and the rise of next-generation sequencing (NGS) technology have facilitated further progress in research on eccDNA. In 2012, Shibata Y et al. identified tens of thousands of short extrachromosomal circular DNA in mouse tissues and mouse and human cell lines, which are 100–400 bp long, derived from unique nonrepetitive sequences and rich in the 5'-untranslated region of genes, exons and CpG islands [25]. Because these extrachromosomal circular DNAs were not consistent with the abovementioned DMs, they were called microDNA. In 2014, Nathanson et al. identified eccDNA that carried the EGFRvIII mutant gene in glioma cells and could mediate resistance to EGFR inhibitors [26]. In 2017, Verhaak et al. analysed the whole genome of 2572 cell lines derived from 17 tumours and found that more than half of the human tumours in the study contained eccDNA [27]. These eccDNAs often carried tumour-driving genes. This was the first time that researchers recognized that eccDNA generally plays an important role in cancers. In 2018, Møller HD et al. isolated more than 100,000 types of eccDNA from muscle and blood cells of healthy people, and most of this eccDNA carried complete gene or gene fragments, often less than 25 kb in length [28]. This report suggested that eccDNA may be ubiquitous in various cells of the human body. In 2019, Wu S et al. found that there are significant chromosomal open states and more distant interactions in eccDNA, which improved researchers' understanding of the mechanism of action of eccDNA [29]. At the same time, Morton AR et al. analysed the chromatin structure and enhancer state of eccDNA [30]. The results showed that the enhancer function of the noncoding region in eccDNA plays a role in regulation of the expression of oncogenes carried by eccDNA, which further indicates the special chromatin structure and function of eccDNA.

## The biological characteristics of eccDNA

### Small polydispersed circular DNA (spcDNA)

In 1965, Hotta et al. proposed that there are significant differences in the size of DNA in the cells of higher plants and animals and that DNA in mammalian cells can exist in a circular form [4]. Subsequently, in HeLa cells, researchers found a large number of closed circles of DNA of unequal sizes [17]. In 1972, Smith et al. studied these circular DNAs and found that their size ranged from 0.2 to 2.0 μm; for the first time, they officially named them spcDNA [18]. At that time, spcDNA and eccDNA were almost completely interchangeable words, but with the discovery of other types of eccDNA, especially as an increasing number of DMs were observed under an optical microscope, this practice became increasingly inappropriate. At present, spcDNA refers to eccDNAs of several hundred to several thousand bp with a size of 0.2–2.0 μm, and huge differences exist with regard to their number in various cells, ranging from less than one hundred copies per cell to thousands of copies per cell [31].

The modes of spcDNA formation are not clear at present, but because of the huge differences in size, amount, sequence content and organization, it is reasonable to believe that there are many different modes of formation in cells. Studies have shown that spcDNA is mainly derived from repetitive sequences in the genome, and the presence of high levels of direct tandem repetitive sequences is conducive to the production of spcDNA in early embryonic cells [7, 32]. Regarding the formation mechanism of spcDNA, at present, the main theories include nonhomologous recombination, homologous recombination and retrotransposition [18, 33–38]. In addition, some researchers have proposed a new pathway that relies on DNA ligase IV [39]. However, most of the currently known spcDNA formation mechanisms are still speculative. In one cell, there may be several simultaneously occurring mechanisms for producing spcDNA, but homologous chromosome recombination is still the preferred method.

The biological function of spcDNA is still unclear. Although it is an important part of eccDNA, its quantity in human cells is limited [7]. Studies have shown that spcDNA is often enriched in genetically unstable cells, such as tumour cells (HeLa cell line) and cells in tumour tissues (colon carcinoma) and genomic instability disease (Fanconi’s anaemia) [7, 40]. Therefore, we believe that spcDNA must be related to human genetic instability. Autonomous spcDNA replication may play an active role in the initiation and enhancement of genomic instability, leading to changes in chromosome structure and gene expression patterns [7].

### Telomeric circles (T-circles)

The first thing to make clear is that the telomeric loop (T-loop) and telomeric circle (T-circle) are two completely different structures. Telomeres are repetitive nucleotide fragments that exist at the end of linear DNA and protect DNA from degradation and fusion. They have relatively conserved sequences, such as TTAGG/CCCTAA in mammals [41]. A telomeric array is a tract of tandemly repeated sequences at the end of linear DNA, usually ending with a single-stranded overhang, and is a prerequisite for the formation of higher-order structures such as T-loops. T-loops are generated by invasion of a telomeric overhang into the duplex region of telomeric arrays, and T-circles are extrachromosomal circular DNA molecules composed of telomeric arrays [8]. In 1995, researchers discovered for the first time that there are a variable number (up to 12) of long (738 bp) tandem terminal repeats at the end of mitochondrial DNA in yeast [42]. Later, this repetitive sequence with a size equivalent to a multiple of 738 bp was found to have an open circular structure and was officially named a T-circle [43].

In a variety of cell lines, researchers have found that the size and distribution of T-circles from alternative lengthening of telomeres (ALT) in cells are closely related to T-loops isolated in the same cell line, indicating that T-circles are dissociated from T-loops [44, 45]. Later, three hypotheses regarding the formation mechanism of T-circles were proposed: (1) mtDNA and hypersuppressive-like T-circles can coexist in strictly aerobic petite-negative organisms, and eventually, the two recombine, which may lead to the formation of a linear DNA genome with T-arrays at the ends. (2) T-circles may come from intrastrand recombination of units within a pre-existing intrachromosomal array of tandem repeats. (3) T-circles may be produced by reverse transcription of a type of RNA, which serves as a template for first-strand cDNA synthesis, followed by template degradation, synthesis of the second cDNA strand and ligation of nicks [8]. In addition, studies have shown that multiple proteins, including telomeric repeat binding factor 2 (TRF2), WRN recQ-like helicase (WRN), BLM recQ-like helicase (BLM) and regulator of telomere elongation helicase (RTEL), may be involved in the formation of T-circles [46–49].

The T-circle is currently believed to have a similar effect on telomerase, which can add new telomere repeats to the ends of chromosomes to compensate for the shortening of telomeres caused by DNA replication [50, 51]. T-circles have been shown to play an important role in ALT and were first discovered in yeast [43, 52]. Since t T-circles can be used as a template for telomere DNA rolling-circle replication, they can effectively extend telomeres.

### MicroDNA

MicroDNA was first discovered in 2012. In mouse tissues and mouse and human cell lines, Shibata Y et al. identified a new type of eccDNA with a size of 100–400 bp that was derived from unique nonrepetitive sequences. It was enriched in the 5'-UTR, exons and CpG islands and officially named microDNA [25]. Further research has shown that most microDNA is derived from areas with high gene density, GC content, and exon density; from promoters with activating chromatin modifications; and in sperm from the 5'-UTR of full-length LINE-1 elements but is depleted from lamin-associated heterochromatin [9].

The microDNA formation mechanism is currently unclear. Dillon et al. proposed the following hypothesis: (1) In the process of DNA replication, polymerase slippage at the short direct repeat sequence will result in the formation of DNA loops on the product or template strand, while the excision of the loop and subsequent ligation may result in the formation of ss microDNA. (2) DNA breaks or stalls allow newly synthesized nascent DNA strands to circulate with the help of short tiny stretches of homology on the template. Connecting such loops will form ss microDNA. (3) Two DNA double-strand breaks, followed by microhomology-mediated circularization of released fragments, may lead to the generation of ds microDNA [9].

MicroDNA is very small, which makes it difficult for the molecule to carry complete genes to encode proteins. Paulsen T et al. found that microDNA may regulate gene expression by producing regulatory small RNAs [53]. They tested the functions of microDNA by simulating known microDNA sequences and found that they can express functional small regulatory RNAs, including microRNAs and new si-like RNAs. The microDNA carrying the miRNA gene forms a transcript, which is processed into a mature miRNA molecule through the endogenous RNA interference pathway to inhibit an endogenous mRNA target. In addition, microDNA containing exon sequences inhibits endogenous genes derived from microDNA by forming new si-like RNAs.

### Extrachromosomal DNA (ecDNA)

As early as 1965, DMs were first found in metaphase neuroblastoma cells [5] and subsequently in various types of cancer [54–58]. According to the Mitelman database, the total frequency of DMs in primary cancers is 1.4% [59], and the frequency of DMs in malignant tumours is much higher than that in benign tumours [60]. However, the total frequency of DMs in the database is much lower than that in previous reports, which may be due to flaws in the concept of DMs. With the advancement and application of molecular biology research technology, Mischel et al. proposed that the scope of ecDNA must be expanded [27] because it can exist in dimeric form (such as DMs) or in monomeric form, while DMs only occupy 30% of the total amount of ecDNA [29]. Currently, the term ecDNA refers to all DNA that is extrachromosomal, circular, and 1–3 Mb in size.

**Fig. 1:**
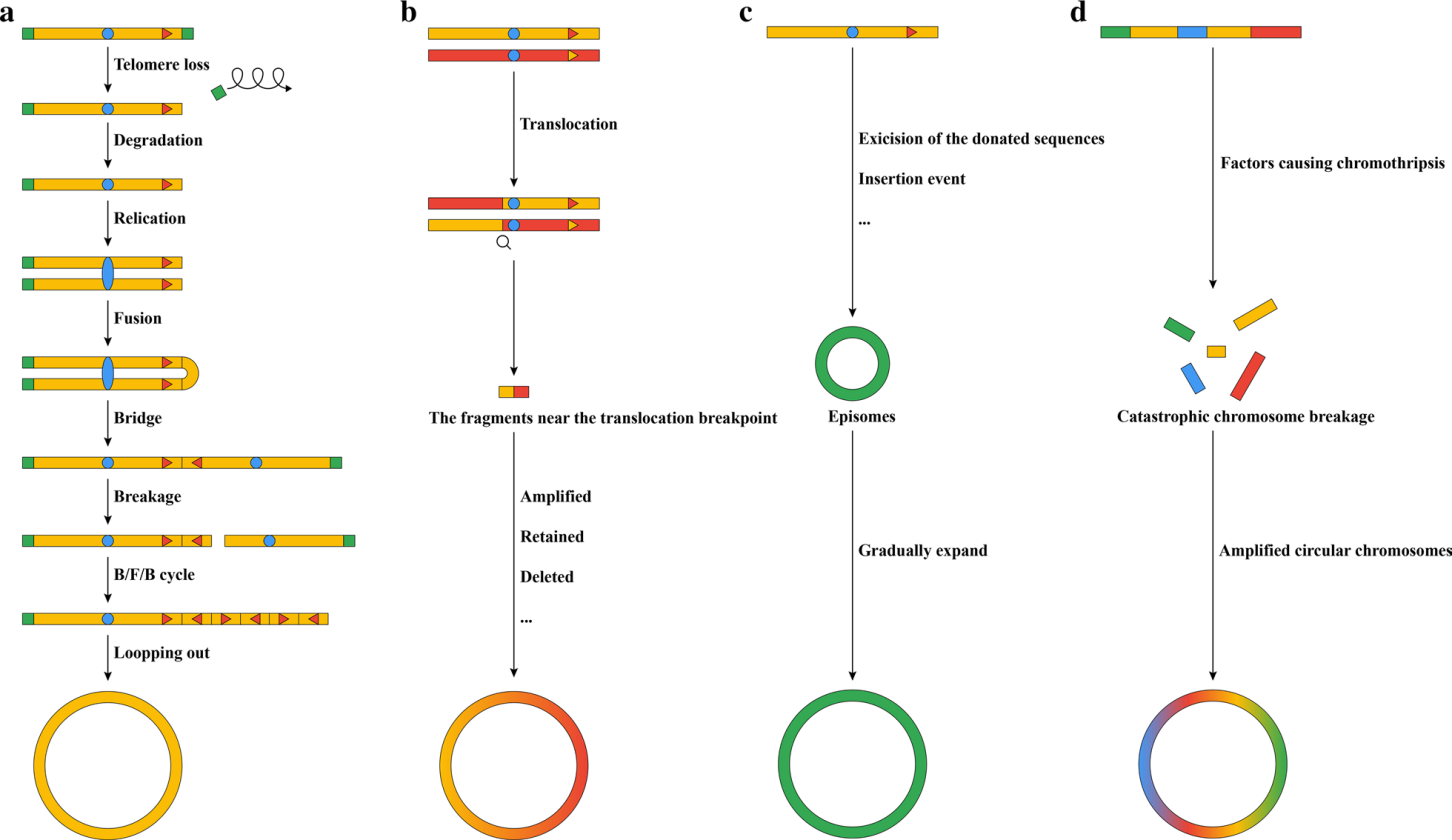
Four models of ecDNA formation. Four models have been proposed to explain the formation of ecDNA. **A** The breakage/fusion/bridge (B/F/B) cycle. **B** Translocation-excision-deletion-amplification mechanism. **C** Episome model. **D** Chromothripsis

The formation of ecDNA is diverse and complex, and four models have been proposed to explain ecDNA formation. (1) The breakage/fusion/bridge (B/F/B) cycle (Fig. 1A) is one of the most classic models. This hypothesis was first proposed by McClintock B in 1951 [61]. It is one of the chromosomal instability mechanisms responsible for characteristic mitotic abnormalities and the occurrence of specific classes of genomic rearrangements. This cycle is caused by fusion of broken chromosome ends or erosion of telomere ends formed by double-stranded DNA breaks (DSBs). Subsequent formation of dicentric chromosomes, anaphase bridges and their resolution is a self-continuous process because every time an anaphase bridge is resolved, a new chromosome break is introduced. This leads to inverted duplications in one daughter cell and terminal deletions in the other daughter cell [62]. Depending on the location and size of the break, the B/F/B cycle will produce various chromosomal rearrangements, including gene amplification, large duplication, large deletion and ecDNA [63]. (2) The translocation-excision-deletion-amplification mechanism (Fig. 1B) was proposed after Barr et al. studied alveolar rhabdomyosarcoma and found that translocation and amplification events are synergistic and can have powerful carcinogenic activity [64]. This mechanism was further summarized as a translocation-excision-deletion-amplification mechanism [65]. In this model, gene rearrangements occur near the translocation site. The fragments near the translocation breakpoint are amplified, retained or deleted, which may lead to formation of ecDNA. The model also verified the formation of fusion genes, such as paired box 3 (PAX3)- Forkhead Box O1 (FOXO1), paired box 7 (PAX7)- FOXO1, and high mobility group AT-hook 2 (HMGA2)-MDM2 proto-oncogene (MDM2) [64, 66]. (3) The episome model (Fig. 1C) was developed in 1987, when Carroll et al. discovered that there is a covalently closed circle of 250 ~ 300 kb outside the chromosomes that can mediate gene amplification and the genesis of minute chromosomes [67]. They believed that this episome was formed by excision of the donated sequences along with a nearby replication origin or that an origin was created by the insertion event itself. Later, the researchers discovered that these episomes can gradually expand until they become DMs, and then, the DMs can be integrated into chromosomes [68]. The formation of DMs in leukaemia and neuroblastoma can also be explained by the episomal model [69, 70]. (4) The concept of chromothripsis (Fig. 1D) was identified in 2011, when Stephens et al. discovered a phenomenon in which tens to hundreds of genome rearrangements occurred in a one-time cell crisis. The rearrangement involving one or several chromosomes crosses back and forth in related regions, generating frequent oscillations between the two copy number states, a process that researchers call chromothripsis [71]. When local chromosome breakage and repair occur in a one-off disaster, chromothripsis will scar the genome. As a result of this process, large-scale DNA rearrangements affecting one or several chromosomes can be detected [72]. This special phenomenon has been found in a variety of tumours, including pancreatic cancer, neuroblastoma, prostate cancer, paediatric medulloblastoma and small cell lung cancer [73–77].

The size of ecDNA is large enough to carry a complete gene, and thus, its main biological function is gene transcription. Studies have shown that ecDNA is preferentially located in the perinuclear region during the G1 phase of the cell cycle, which is consistent with its location during the M phase. Once DNA replication starts, it quickly shifts into the nucleus and replicates semisynchronously in early S phase [78, 79]. Chromatin is a layered structure in which DNA and histones are compressed. This complex structure limits its ability to be transcribed [80]. However, when amplified on ecDNA, oncogene amplification and tumour heterogeneity may be significantly higher than when amplified within chromosomes, which may be related to the unique circular structure of ecDNA [27]. It has been confirmed that ecDNA plays an important role in a variety of cancer types by amplifying genes related to carcinogenesis and drug resistance. In addition, ecDNA is also related to ageing and gene compensation [81, 82].

## Main eccDNA research methods

### How can a rough analysis of eccDNA be achieved?

#### Optical microscopy

Due to the relatively large molecular weight of some eccDNAs, ordinary DNA dyes and optical microscopy can be used to observe extrachromosomal DNA signals in cells in the M phase, some of which are eccDNA signals. The ultrahigh-resolution microscope technology developed in recent years can also be used for eccDNA imaging. Super-resolution three-dimensional structured illumination microscopy (3D-SIM) has been used to perform imaging analysis of eccDNA [29].

#### Electron microscopy

Due to the limitation imposed by the resolution of optical microscopes, it is difficult to observe and analyse the fine structure of eccDNA. Therefore, researchers have turned to electron microscopy to solve this problem. Electron microscopy has enabled significant contributions to be made in research on eccDNA. Both scanning electron microscopy (SEM) and transmission electron microscopy (TEM) can be used for imaging eccDNA. Some researchers have also used a photoelectric technique that superimposes confocal light microscopy and SEM signals in the same field of view to perform eccDNA imaging [29].

#### Density gradient centrifugation

Caesium chloride (CsCl) density gradient centrifugation was an important technical method for early DNA research. Since its application in separating ^14^N DNA and ^15^N DNA in 1958, this method has been widely used [83]. Of course, it can also be used to separate circular DNA molecules. However, due to the demand for large samples and the low abundance of eccDNA, this method is currently less commonly used.

#### Assay of transposase-accessible chromatin with visualization (ATAC-see)

ATAC-see is a transposase-mediated imaging technology that uses direct in situ imaging, cell sorting and deep sequencing of accessible genomes to reveal the identity of imaged elements. Combining this technique with flow cytometry enables automated quantitative analysis, and cell separation is expected to be a function of chromatin accessibility, which reveals the dependence of chromatin accessibility on the cell cycle, especially in G1 phase [84]. Currently, this method has been used to visualize ecDNA accessibility in metaphase chromatin [29].

#### Fluorescence in situ hybridization (FISH)

FISH was first proposed in 1969 [85, 86]. It is a method for locating nucleic acid targets in fixed cells for cytogenetics or gene expression research. It relies on fluorescently labelled DNA or RNA probes to count and locate specific genes or regions on chromosomes, detect mutations or analyse the temporal and spatial expression of genes. Using eccDNA fluorescent probes to detect eccDNA in cell samples, FISH is an important tool for more effective observation of known eccDNA.

#### Third-generation sequencing (TGS)

The short read length of NGS technology can cause errors and ambiguities in read mapping [87, 88]. The rise of TGS, including nanopore, heliscope, and single-molecule, real-time (SMAT) sequencing, has solved these problems [89–91]. ecDNA is often large and may carry sequences from multiple chromatin sources. Therefore, it is difficult to completely construct a full-length ecDNA sequence. TGS technology has significantly improved the problem of molecular read length in sequencing and may be of great help in identification of full-length ecDNA sequences.

### How to verify that eccDNA is circular?

Direct observation of eccDNA with a microscope is enough to simply and clearly confirm its circular structure, but researchers can also choose two-dimensional electrophoresis (2-DE) to indirectly verify that the structure of eccDNA is circular. 2-DE was first proposed in 1970 [92]. It is a widely used molecular biology research technique that uses the difference in isoelectric point and molecular weight between substances to achieve high resolution through electrophoresis in two directions [93]. Since the structures of circular DNA and linear DNA are essentially different, they can be separated by vertical 2-DE.

### Advanced eccDNA structure research methods

#### Circle-Sequencing (Circle-Seq)

**Fig. 2:**
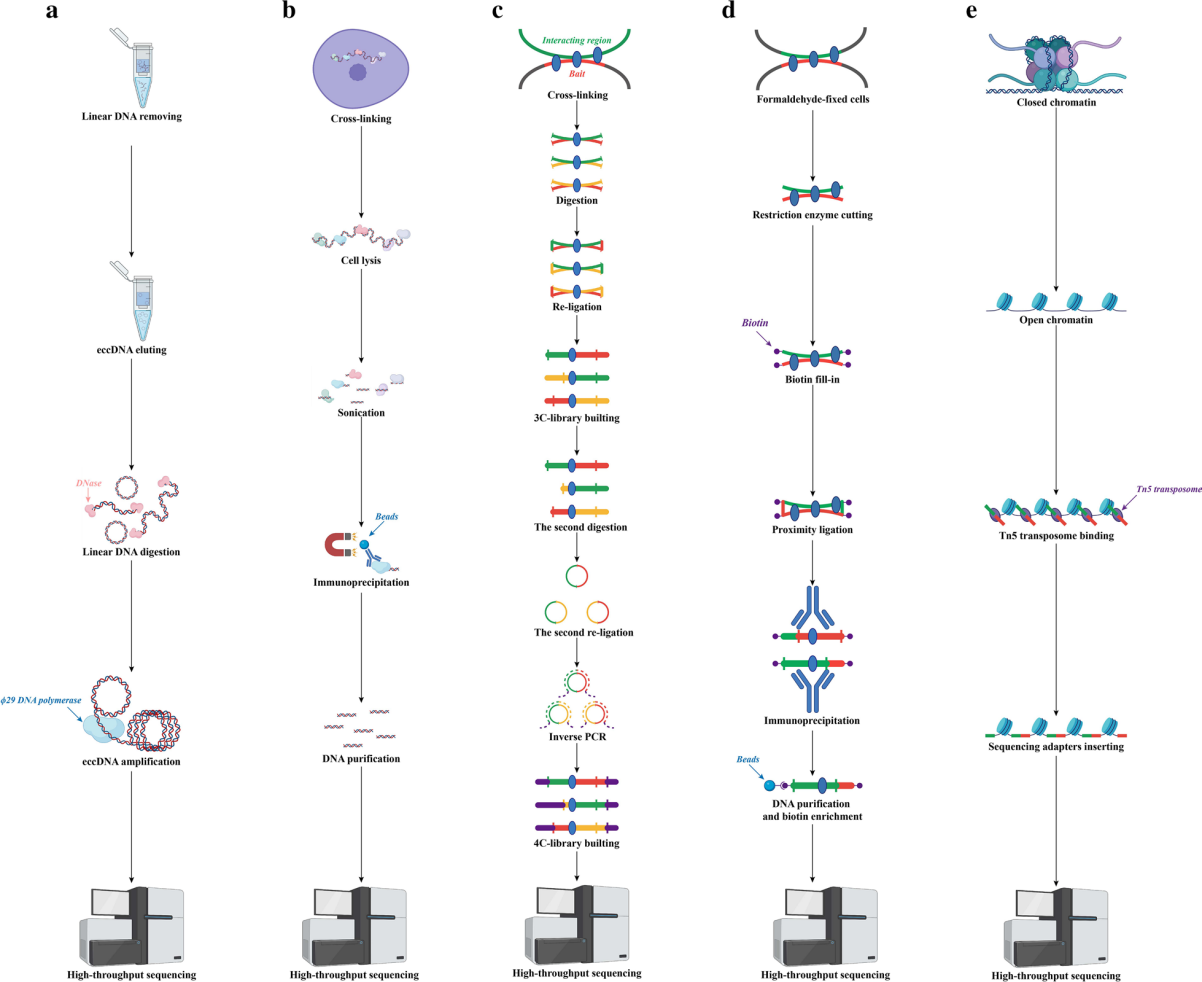
Advanced eccDNA structure research methods. Five research methods can be used to study the advanced structure of eccDNA. **A** Circle-Sequencing (Circle-Seq). **B** Chromatin Immunoprecipitation Sequencing (ChIP-seq). **C** Circular chromosome conformation capture combined with high-throughput sequencing (4C-seq). **D** Proximity ligation-assisted ChIP-seq (PLAC-seq). **E** Assay for Targeting Accessible Chromatin with high-throughput sequencing (ATAC-seq)

Based on mature prokaryotic plasmid purification and sequencing technology, researchers have developed a sensitive, large-scale circular DNA enrichment and detection method: circle-Seq [19, 94, 95]. In this method, purified circular DNA is first purified from broken cells using a circular DNA column, and then, the remaining linear DNA is digested by plasmid-safe ATP-dependent DNase promoted by NotI endonuclease. Then, ϕ29 DNA polymerase is used to achieve rolling circle amplification of circular DNA, and finally, high-throughput sequencing can be implemented to detect eccDNA (Fig. 2A).

#### Chromatin Immunoprecipitation Sequencing (ChIP-seq)

ChIP-Seq technology, which combines chromatin immunoprecipitation (ChIP) with NGS technology, can efficiently detect DNA segments in the whole genome that interact with histones and transcription factors. The principle of ChIP-Seq is as follows: First, the DNA fragments bound by the target protein are specifically enriched via ChIP and purified, library construction is performed, and then, the enriched DNA fragments are subjected to NGS. Researchers have accurately located millions of sequence tags on the genome to obtain information about the DNA segments throughout the genome that interact with histones and transcription factors [96, 97]. When studying plasma eccDNA, the researchers found the presence of abundant monomethylation of histone H3 at lysine 4 (H3K4me1) and acetylation of histone H3 at lysine 27 (H3K27ac) modifications, which suggests that ChIP-seq may be an important tool that can be helpful in the study of eccDNA [98] (Fig. 2B).

#### Circular chromosome conformation capture combined with high-throughput sequencing (4C-seq)

4C-seq is a method for analysing the high-level structure of human chromatin. The main principle is to use formaldehyde to cross-link DNA and DNA-bound proteins and then digest and connect cells. After the crosslinking is reversed, the DNA is purified. Finally, a second round of digestion and ligation is performed to generate a 4C library. After circularizing and amplifying the 4C library, the composition of the 4C-seq PCR product can be determined using NGS. By using customized data analysis methods, sequencing results can be mapped to the genome and transformed into the genome-wide interaction pattern of the initially selected bait [99]. 4C-seq can more clearly analyse the interaction of the same segment of chromatin with multiple chromatins [100]. Currently, researchers have used 4C-seq to study ecDNA [29] (Fig. 2C).

#### Proximity ligation-assisted ChIP-seq (PLAC-seq)

PLAC-seq is a fast, sensitive and economical method to map long-distance chromatin interactions in mammalian cells. It is mainly used to analyse the interactions between different chromatin regions. The principle of the technique is that cells are fixed with formaldehyde and digested. The biotin-labelled nucleotides are then filled in and connected in situ. After lysis of the nucleus, the chromatin is sheared. The soluble chromatin fraction is then immunoprecipitated with specific antibodies modified by anti-transcription factors or histones. Finally, the biotin-labelled DNA corresponding to the linker is enriched, and library preparation and paired-end DNA sequencing are performed [101, 102]. Researchers have used PLAC-seq to map the genome-wide 3D chromatin interactions anchored at DNA bound by histones with H3K27ac modification [29] (Fig. 2D).

#### Assay for targeting accessible chromatin with high-throughput sequencing (ATAC-seq)

ATAC-seq was proposed in 2013 and uses DNA transposase technology to achieve chromatin accessibility analysis [103]. DNA transposase can randomly insert a sequence into the genome. In ATAC-seq, after the nucleus and cytoplasm of the cell or tissue sample are separated, the nuclei are collected separately, and the chromatin in the nucleus is interrupted by transposase. The tightly packed chromatin DNA will not be interrupted by the transposase, while the transposase will cause to random insertions and interruptions in the chromatin DNA in the open region. These interrupted DNAs are collected for subsequent library building, sequencing, and analysis to obtain information about open chromatin [104] (Fig. 2E).

### eccDNA analysis tools

#### AmpliconArchitect

AmpliconArchitect is a tool that uses whole genome sequencing (WGS) to reconstruct the fine structure of a focal magnification area and has been extensively verified using multiple simulated and real data sets, covering a wide range of coverage and copy numbers [105]. It can reconstruct possible ecDNA and other focal amplicon structures from short read data and allows interactive exploration of alternative structures. The size of ecDNA means that the existing single-molecule assembly is not sufficient for ecDNA reconstruction. Therefore, AmpliconArchitect reconstruction on cheap short read data can be used as a template to guide the assembly of longer single molecule reads. Researchers have used AmpliconArchitect to analyse 68 virus-mediated cancer samples and identified a large number of amplified fragments with special structural features that suggested mixed human viral extrachromosomal DNA. Analysis of AmpliconArchitect reconstructions of multiple pan-cancer datasets suggests that ecDNA may play an important role in creating complex rearrangements and focal magnification observed across cancer subtype spectra.

#### AmpliconReconstructor

ecDNA is the main driving force of focal copy number amplification (FCNA), which enables gene amplification, rapid tumour evolution, and rewiring of regulatory circuits [27]. Deciphering the structure of FCNA is the first step in deciphering its origin mechanism and subsequent biological consequences. The AmpliconReconstructor method can combine the optical mapping (OM) of long fragments (> 150 kb) with NGS to resolve FCNA with single-nucleotide resolution. AmpliconReconstructor uses NGS-derived breakpoint maps and OM brackets to generate high-fidelity reconstructions. After verifying its performance through a variety of simulation strategies, researchers used AmpliconReconstructor to reconstruct CNAs in seven tumour cell lines, revealing the complex structure of ecDNA, break-fusion bridges and other complex rearrangements. By reconstructing the rearrangement signatures related to the FCNA generation mechanism, AmpliconReconstructor can help researchers more thoroughly understand the origin of FCNA [106].

#### ecSeg

ecSeg is a U-Net-based platform used to automatically classify 4',6-diamidino-2-phenylindole (DAPI) signals, identify and quantify ecDNA, and combine FISH data to clarify the location of oncogene amplification on ecDNA and chromosomes. ecSeg divides each image pixel in DAPI- and FISH-stained images into one of the following categories: cytoplasm, nucleus, chromosome and ecDNA and calculates the connected components of ecDNA pixels to calibrate and count ecDNA. FISH probes facilitate quantification of their spatial positions in a separate postprocessing step and associate these positions with ecDNA and chromosomes [107].

#### ViFi

ViFi is a tool for detecting viral integration and fusion mRNA sequences from NGS data [108]. Unlike the standard method that uses reference-based read mapping to identify virus reads, ViFi uses reference-based read mapping and a phylogeny-based method to identify virus reads. ViFi also incorporates the mappability score of the readings to filter out false positives and integrate detection. The final result is a tool that can accurately detect integrated viruses even if the virus is a highly mutated or new strain.

#### ECdetect

ECdetect is a semiautomatic image analysis software package for cytogenetic analysis and is used in combination with whole-genome sequencing [109]. ECdetect uses the fluorescent dye DAPI to quantify ecDNA in metaphase cells. The software has been used to quantify ecDNA in 2,572 cells from various cancer types, including 72 primary cancer cell samples, 10 immortalized tumour cell lines and 8 noncancer control cell lines [27] (Table 2).

**Table 2:** The details of eccDNA analysis tools

Name	Characteristics	Website
AmpliconArchitect	It is a tool which can reconstruct the structure of focally amplified regions in a cancer sample using whole genome sequence short paired-end data	https://github.com/virajbdeshpande/AmpliconArchitect
AmpliconReconstructor	It produces an ordering and orientation of graph segments, with fine-structure information from the breakpoint graph embedded into the large-scale reconstructions and as output, it reports large-scale reconstructions of fCNA amplicons	https://github.com/jluebeck/AmpliconReconstructor
EcSeg	It is a tool used to quantify ecDNA in metaphase images. It also has an extension to analyze FISH probes	https://github.com/ucrajkumar/ecseg
ViFi	It is a tool for detecting viral integration and fusion mRNA sequences from Next Generation Sequencing data. It uses both reference-based read mapping and a phylogenetic-based approach to identify viral reads and incorporates mappability scores of the reads to filter out false positive integration detection	https://github.com/namphuon/ViFi
ECdetect	It performs image pre-segmentation to distinguish between chromosomal and non-chromosomal structures, and calculates the interest (ROI) of the search area of ecDNA. The output of the software includes the original image covered with ecDNA detection, the number of ecDNA found, and their coordinates in the image	ECdetect will be available upon request

## The role of eccDNA in cancer

**Fig. 3:**
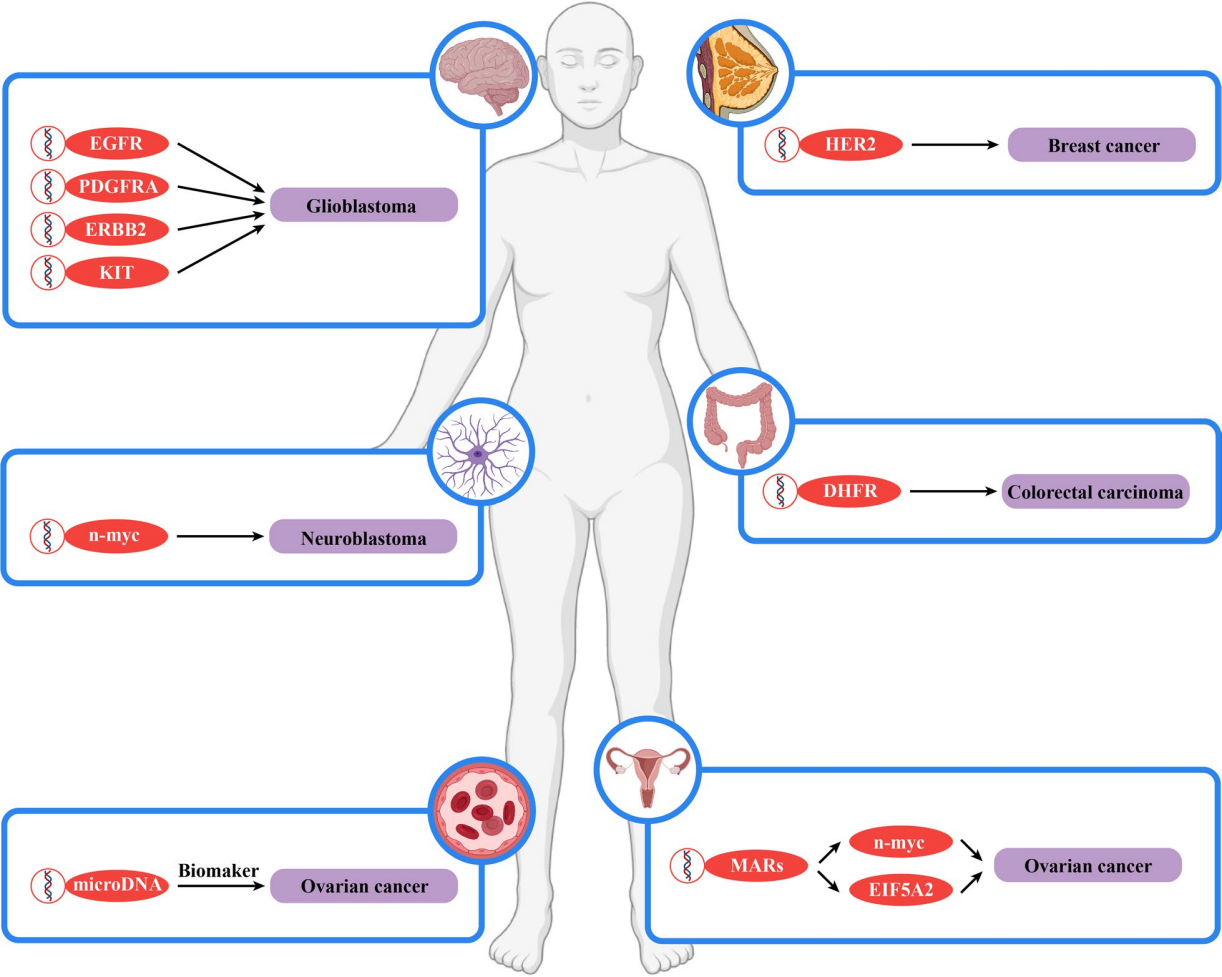
The roles of ecDNA in cancer progression. In contrast to microDNA, which regulates gene expression, and eccDNA, which can serve as a biomarker to aid in cancer diagnosis and prognosis prediction, ecDNA can also carry oncogenes, and amplification of these oncogenes affects cancer occurrence, development and drug resistance

Many studies have shown that eccDNA plays an important role in a variety of cancer types. For example, microDNA can regulate gene expression, eccDNA can serve as an assistant diagnosis and prognosis prediction biomarker, and ecDNA can carry oncogenes and facilitate their amplification (Table 3, Fig. 3).

**Table 3:** The roles of ecDNA in cancer progression

Cancer	Oncogene amplification via ecDNA	The connection between ecDNA and tumorigenesis
Neuroblastoma	n-myc	Remodeling the chromosomal genome, amplifying n-myc, promoting the expression of TERT and inhibiting the expression of DCLK1
Glioblastoma	EGFR, PDGFRA, ERBB2, KIT	Amplifying a variety of oncogenes, affecting tumor invasiveness, radiation resistance and drug resistance
Colorectal carcinoma	DHFR	Amplifying DHFR to develop drug resistance
Ovarian cancer	n-myc, EIF5A2	Influencing the expression of ecDNA through MARS
Breast cancer	HER2	Amplifying HER2 to affect tumor drug resistance

### Regulation of gene expression

Although the size of microDNA restricts its ability to carry complete genes to encode functional proteins, it can express functional small regulatory RNAs, such as microRNAs and new si-like RNAs, to regulate gene expression. This function has been confirmed in the HCT116, 293A and 293 T cell lines [53]. In addition, researchers have found that in the human lymphoblast cell line, treatment with methotrexate (MTX) and L-asparaginase (ASP) produced more microDNA (particularly with MTX). At the same time, these 180–200 bp microDNAs were not randomly generated but matched with DNA fragments of typical apoptotic cells, suggesting that these microDNAs are generated by programmed cell death or that they participate in regulation of its molecular mechanism [110].

### Biomarkers for diagnosis and prognosis

There have been many studies on circulating free DNA as a tumour biomarker [111]. eccDNA can also be used as a biomarker of various tumours to assist tumour diagnosis and prognosis prediction. Both normal tissues and tumour tissues can release microDNA into the circulation. In a mouse xenograft model of human ovarian cancer, tumour-derived human microDNA was detected in the circulation of mice. The researchers compared the microDNA in paired tumour and normal lung tissue specimens and found that the tumour contained longer microDNA. Compared with the samples obtained from the same patient a few weeks after surgery to remove the tumour, the serum and plasma samples collected before surgery (12 cases of lung cancer and 11 cases of ovarian cancer) were enriched with both longer and higher levels of microDNA [10].

In addition, there are a large number of telomeric repeats in spcDNA molecules (tel-spcDNA) in rodents and human cells. In rodent cells, the level of tel-spcDNA in transformed cells was higher than that in normal cells, and an increase was observed after treatment with the carcinogen *N*-methyl-*N*'-nitro-*N*-nitrosoguanidine (MNNG). tel-spcDNA is also detected in some human tumours and cell lines [112]. Other researchers have also detected spcDNA in tumour cell lines (HeLa), tumour tissues (colon cancer) and fibroblasts from patients with genomic instability disease (Fanconi anaemia). Similarly, induction of spcDNA was observed after treatment with the carcinogen MNNG. This shows that spcDNA can be used as a marker of genomic instability [7]. Regrettably, thus far, there is no research on the use of spcDNA as a biomarker for tumour diagnosis and prognosis judgement.

Meanwhile, some researchers have found that fetal eccDNA in plasma is less methylated than maternal eccDNA. The methylation density of eccDNA was positively correlated with its size. In addition, fetal eccDNA was found to be rapidly cleared from maternal blood after delivery, similar to fetal linear DNA[113]. Unfortunately, the epigenetic nature of eccDNA in cancer has not yet been studied.

### Driving oncogene expression

#### Neuroblastoma

In 1983, Kohl et al. located the N-MYC gene in ecDNA for the first time in neuroblastoma [23]. This was also the first study of ecDNA carrying an oncogene. Subsequently, a comprehensive map of the circularization of extrachromosomal DNA in neuroblastoma was drawn, and it was also verified that the N-MYC gene was amplified on ecDNA in neuroblastoma. Furthermore, the researchers also used short-read and Nanopore sequencing to analyze the structure of N-MYC amplicons, and used ChIP-seq, ATAC-seq and High-throughput chromosome conformation capture (Hi-C) to analyze the chromatin landscape, revealing the two different types of amplicons. The first type collectively amplifies the proximal enhancer driven by noradrenergic core regulatory circuit (CRC). The second type lacks key local enhancers, but contains distal chromosomal fragments harboring CRC-driven enhancers. Therefore, ectopic enhancer hijacking can compensate for the loss of local gene regulatory elements and explain a large part of the structural diversity observed in N-MYC amplification [114]. At the same time, the expression of the oncogene telomerase reverse transcriptase (TERT) was significantly increased due to ecDNA. Interestingly, the expression of the tumour suppressor gene doublecortin-like and CAM kinase-like 1 (DCLK1) was significantly reduced due to ecDNA [115].

#### Glioblastoma

Zhou YH et al. found that EGFR was amplified on ecDNA in glioblastoma and that glioblastoma cells containing ecDNA exhibited stronger invasive properties and radiation resistance [116]. However, the relationship between the two has not been studied in depth. Through single-cell analysis of patient-derived models and clinical samples of glioblastoma patients treated with EGFR tyrosine kinase inhibitors (TKIs), researchers have found that EGFR, c-MYC, N-MYC, and others rely on a high level of ecDNA amplification in glioblastoma cells and that loss of ecDNA that encodes EGFRvIII promotes resistance to TKIs. After stopping TKI treatment, EGFRvIII ecDNA reappeared in a large number in glioblastoma cells [26]. Subsequently, studies have shown that oncogenes, such as EGFR, platelet-derived growth factor receptor alpha (PDGFRA), Erb-B2 receptor tyrosine kinase 2 (ERBB2), and KIT Proto-Oncogene (KIT), are located on ecDNA in glioblastoma and are amplified in large numbers, which plays a role in promoting cancer. At the same time, there are a large number of amplified linked extrachromosomal mutations (ALEMs) [117].

#### Colorectal carcinoma

Through study of colon cancer HT29 cells, researchers found that high-dose MTX treatment often led to a significant increase in the copy number of the DHFR gene depending on ecDNA, which further resulted in HT29 cell resistance to MTX. Discontinuation of the drug resulted in loss of ecDNA carrying the DHFR gene, and the drug resistance of the cells was significantly reduced in the second round of administration. This result indicates that ecDNA can play a guiding role in the second round of colorectal carcinoma (CRC) treatment [118]. In addition, the key nonhomologous end joining (NHEJ) protein DNA-PKcs can promote the amplification of DHFR ecDNA. Inhibition of DNA-PKcs can eliminate DHFR ecDNA and enhance the sensitivity of cells to MTX [119]. Researchers also identified two distinct ecDNA populations in the NCI‐H716 cell line, demonstrated their heterogeneity in cancer cells, and successfully constructed their molecular structure. The distribution of amplicons in the two different ecDNA populations suggests that the multi-step evolutionary model is more suitable for the ecDNA generating module of the NCI‐H716 cell line [120].

#### Ovarian cancer

Researchers found five new matrix attachment regions (MARs) in the 682 kb DM of the human ovarian cancer cell line UACC-1598 through sequencing and bioinformatics analysis. All these MARs interacted with the nuclear matrix in vitro, and all were associated with activating the amplification of oncogenes, mainly N-MYC and eukaryotic translation initiation factor 5A2 (EIF5A2), from ecDNA [121].

#### Breast cancer

Through research on breast cancer tissues and cells, researchers have found that approximately 30% of human epidermal growth factor receptor 2 (HER2)-positive breast cancers exhibit ecDNA HER2 amplification and that this ecDNA carrying the HER2 gene is relatively conserved among resistant tumours, regardless of the resistance mechanism. In various models of resistance to anti-HER2 therapy, the amount of HER2 ecDNA remained almost unchanged, even when resistance was acquired through the loss of HER2 protein expression [122].

## Conclusion

This review has summarized the classification, biological characteristics, formation mechanisms, and physiological functions of various eccDNAs; the current methods and tools for studying eccDNA; and most importantly, the potential role of eccDNA in cancer. Based on size, eccDNA can be roughly divided into spcDNA, telomeric circles, microDNA and ecDNA. They each have their own characteristics, formation process and physiological functions: initiating and enhancing genomic instability, telomerase-like action, producing regulatory small RNA to regulate gene expression, and amplification of genes related to carcinogenesis and drug resistance. Therefore, eccDNA plays a variety of important roles in cancer.

Interestingly, current research seems to show that eccDNA generally plays a role in promoting cancer. In glioma, neuroblastoma, breast cancer, colorectal cancer, and ovarian cancer, ecDNA promotes cancer or tumour cell drug resistance through the amplification of oncogenes. In addition, although there are no definitive research findings that show the specific functions of spcDNA, telomeric circles and microDNA, we are able to use the limited knowledge that is currently available to make the following inferences: spcDNA can enhance genomic instability and lead to cell carcinogenesis.; telomeric circles can constantly repair telomeres and affect cell proliferation and cell cycle progression; and microDNA affects cell gene expression by regulation of small RNA, which leads to cell carcinogenesis. Generally, researchers' understanding of eccDNA is still very limited, and many problems require further study. (1) Is it true that eccDNA only promotes cancer? (2) Is there a mechanism of intercellular eccDNA transmission? As we all know, the tumor microenvironment (TME), including fibroblasts and myofibroblasts, neuroendocrine cells, adipose cells, immune and inflammatory cells, the blood and lymphatic vascular networks, and extracellular matrix (ECM), plays an important role in the initiation, progression and metastasis of tumors [123, 124]. More and more evidences show that the cells in TME are not passive bystanders, but actively participate in and influence various biological processes of cancer cells. At the same time, cancer cells can also affect, even “educate” the cells in TME. In order to maintain this cancer-TME communication mechanism, it is essential to exchange functional molecules between different cell types [125]. Different from the traditional way of intercellular communication, extracellular vesicles (EVs), including microvesicles and exosomes, are getting more and more attention from researchers. EVs contain a variety of substances, such as RNA, proteins, etc., which play an irreplaceable role in intercellular communication [125, 126]. As a DNA molecule independent of chromosomes, eccDNA undoubtedly has a greater advantage over small RNAs: eccDNA theoretically has a longer half-life, a more stable biological structure, and can carry more genetic information, so more information can be transmitted in intercellular communication. Small RNAs have a short half-life, are not stable enough, and are easily degraded by various enzymes. Their limited size also implies that the genetic information they carry is extremely scarce. More importantly, most small RNAs do not have the ability to independently encode proteins and can only play an indirect regulatory role, while ecDNA has complete genes and can independently encode proteins. eccDNA may be transmitted between cells through EVs, thereby forming a more complex biological network and promoting the development of tumor heterogeneity. (3) Furthermore, more and more evidences show that microbe are widespread in various types of tumors [127]. Researchers have discovered that bacteria are present in every type of tumor from the brain to the bone, and different types of tumors have their own unique microbiomes. Among them, the bacterial community displayed by breast tumors is particularly rich and diverse: the number of bacteria is the largest and the variety is the largest [128, 129]. The above results suggest that the microbiome may also be part of TME. At the same time, studies have also found that bacteria that invade tumor cells will present their protein fragments to the surface of tumor cells, and then be recognized by the immune system. This discovery may be used in cancer immunotherapy [130, 131]. Can the tumor-resident bacteria found in the tumor communicate with each other? What kind of communication is there between bacteria and cancer cells? Current studies have shown that eccDNA exists in both bacteria and cancer cells, and eccDNA is completely possible to be secreted out of bacteria. Therefore, we can speculate that eccDNA may be involved in the communication between bacteria and bacteria and between bacteria and cells in TME, thus affecting the development of cancer and promoting tumor heterogeneity. (4) At present, there is almost no research on targeted cancer therapy related to eccDNA. Tumour-targeted therapy is considered to be an excellent approach for tumour therapy. Effective target selection is the key to targeted therapy. However, one possible reason that the development of targeted therapy remains a challenge is because the function of eccDNA has not yet been fully elucidated; this is especially true for ecDNA, which has the capacity to carry a complete gene sequence. In addition, are there any splicing hotspots that form eccDNA? Does eccDNA have a significant influence or exert feedback regulation on the original genomic DNA? How can eccDNA be enriched or lost in the process of tumorigenesis and development? We believe that with the continuous progress in research, answers to these questions can be found.

eccDNA has been discussed for decades. The rapid development of eccDNA research not only marks a significant advancement in molecular biology but also subverts our traditional understanding of DNA. It has helped to further reveal the biological mechanism of tumorigenesis and may provide new targets for tumour therapy. Undoubtedly, there is still a long way to go before a full understanding of eccDNA is achieved. However, we believe that future research on eccDNA will focus on the following goals: first, to further clarify the specific biological functions of various eccDNA types, study the specific mechanisms of eccDNA in intercellular communication and even tumor microbiome in TME, and construct a complex regulatory network model of eccDNA in a single cancer type; second, to expand the sample size of clinical cases and to consider eccDNA as a factor in the early diagnosis of tumours and patient prognosis; third, to develop targeted cancer treatments related to eccDNA to provide new potential options for cancer treatment.

## Data Availability

Not applicable.

## Abbreviations

eccDNA: Extrachromosomal circular DNA
bp: Base pair
spcDNA: Small polydispersed circular DNA
t-circle: Telomeric circle
ecDNA: Extrachromosomal DNA
DM: Double minutes
DHFR: Dyhidrofolatereductase
EGFR: Epidermal growth factor receptor
NGS: Next generation sequencing
T-loop: Telomeric loop
ALT: Alternative lengthening of telomeres
TRF2: Telomeric repeat binding factor 2
WRN: WRN recQ like helicase
BLM: BLM recQ like helicase
RTEL: Regulator of telomere elongation helicase
B/F/B: The breakage/fusion/bridge cycle
DSB: Double-stranded DNA breaks
PAX3: Paired box 3
FOXO1: Forkhead box O1
PAX7: Paired box 7
HMGA2: High mobility group AT-hook 2
MDM2: MDM2 proto-oncogene
3D-SIM: Three-dimensional structured illumination microscopy
SEM: Scanning electron microscope
TEM: Transmission electron microscope
CsCl: Cesium chloride
ATAC-see: Assay of transposase-accessible chromatin with visualization
FISH: Fluorescence in situ hybridization
TGS: Third-generation sequencing
SMAT: Single-molecule, real-time sequencing
2-DE: Two-dimensional electrophoresis
Circle-Seq: Circle-Sequencing
ChIP-seq: Chromatin Immunoprecipitation Sequencing
H3K4me1: Monomethylation of histone H3 at lysine 4
H3K27ac: Acetylation of histone H3 at lysine 27
4C-seq: Circular chromosome conformation capture combined with high-throughput sequencing
PLAC-seq: Proximity ligation-assisted ChIP-seq
ATAC-seq: Assay for Targeting Accessible-Chromatin with high-throughput sequencing
WGS: Whole genome sequencing
FCNA: Focal copy number amplification
OM: Optical mapping
DAPI: 4', 6-Diamidino-2-phenylindole
MTX: Methotrexate
ASP: l-asparaginase
tel-spcDNA: Telomeric repeats in spcDNA
MNNG: M-methyl-*N*'-nitro-*N*-nitrosoguanidine
Hi-C: High-throughput chromosome conformation capture
CRC: Core regulatory circuit
TERT: Telomerase reverse transcriptase
TKIs: Tyrosine kinase inhibitors
PDGFRA: Platelet-derived growth factor receptor alpha
ERBB2: Erb-B2 receptor tyrosine kinase 2
KIT: KIT Proto-Oncogene
ALEMs: Amplified linked extrachromosomal mutations
CRC: Colorectal carcinoma
MARs: Matrix attachment regions
EIF5A2: Eukaryotic translation initiation factor 5A2
HER2: Human epidermal growthfactor receptor 2
TME: Tumor microenvironment
ECM: Extracellular matrix
EVs: Extracellular vesicles
